# Supplementary material for: Rhodium nanocrystals on porous graphdiyne for electrocatalytic hydrogen evolution from saline water
Source: Nat Commun. 2022 Sep 5;13:5227. doi: 10.1038/s41467-022-32937-2 (PMC9445080; doi:10.1038/s41467-022-32937-2)
Supplement: Supplementary file 1 — Supplementary Information [file 41467_2022_32937_MOESM1_ESM.pdf]

Supplementary Information

Rhodium nanocrystals on porous graphdiyne for electrocatalytic hydrogen evolution from saline water

Yang Gao[1], Yurui Xue[1,2]*, Lu Qi[2], Chengyu Xing[1], Xuchen Zheng[1,3], Feng He[1]* and Yuliang Li[1,3]*

[1]CAS Key Laboratory of Organic Solids, Institute of Chemistry, Chinese Academy of Sciences, Beijing 100190, P. R. China.

[2]Science Center for Material Creation and Energy Conversion, Institute of Frontier and Interdisciplinary Science, School of Chemistry and Chemical Engineering, Shandong University, Jinan 250100, P. R. China.

[3]University of Chinese Academy of Sciences, Beijing 100049, P. R. China.

*E-mails: yrxue@sdu.edu.cn, hefeng2018@iccas.ac.cn, ylli@iccas.ac.cn.

Supplementary Figures

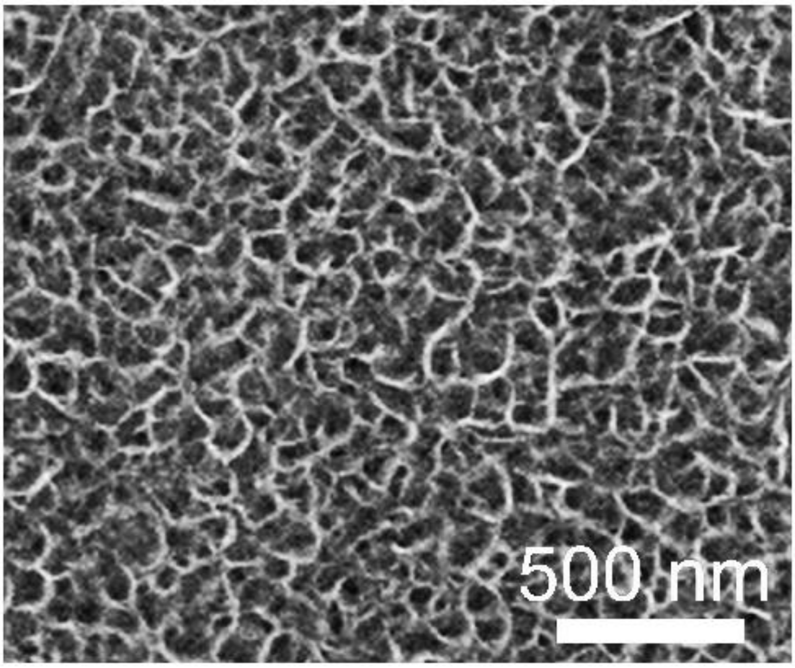

Supplementary Fig. 1 | Morphological characterization. High-magnification SEM image of GDY nanosheets array.

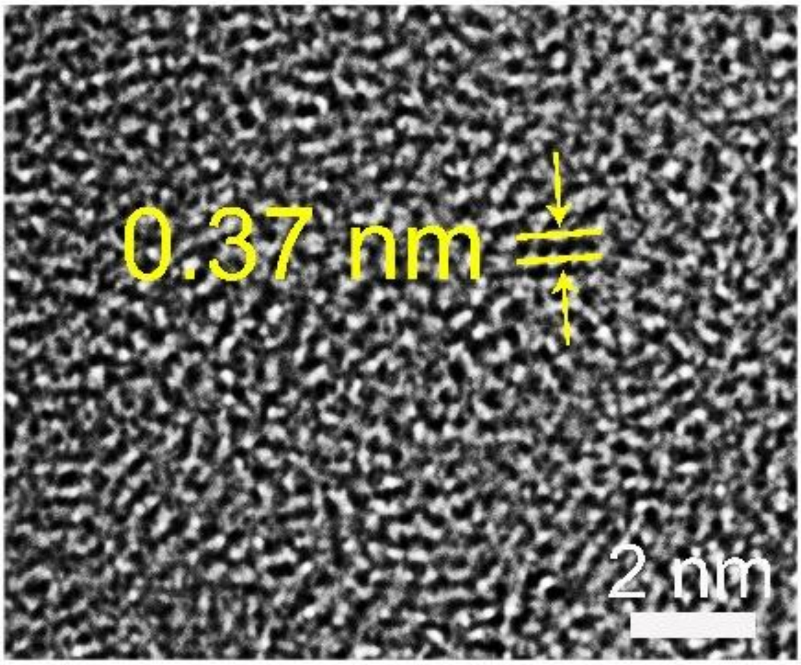

Supplementary Fig. 2 | Morphological characterization. High-magnification TEM image of GDY nanosheet.

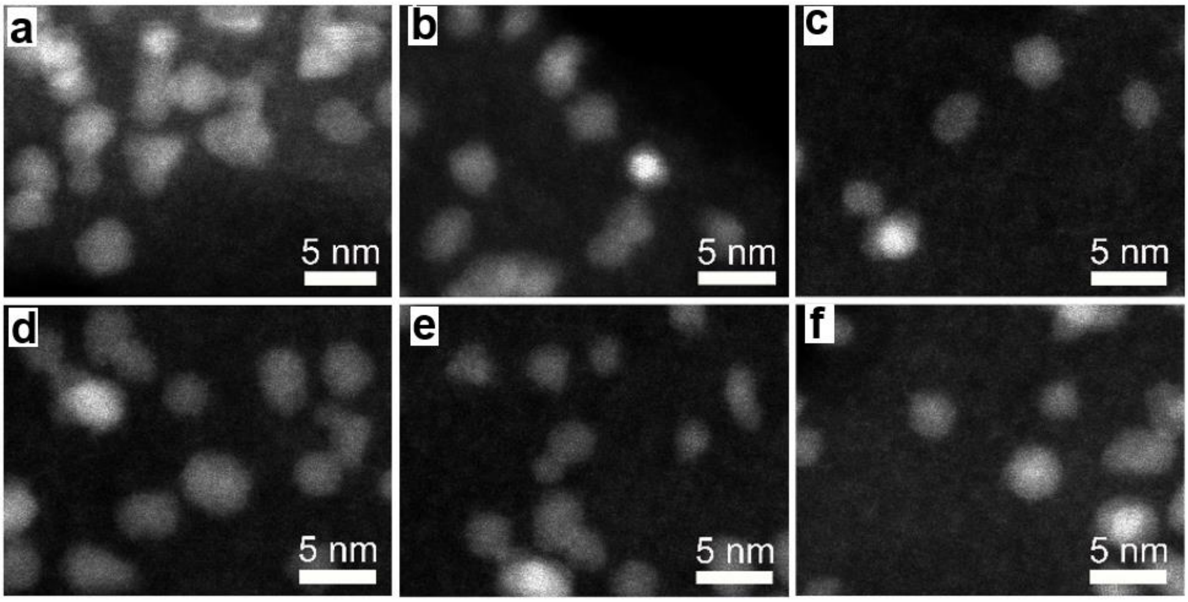

Supplementary Fig. 3 | Morphological characterization. a-f High-magnification HAADF-STEM images of Rh/GDY.

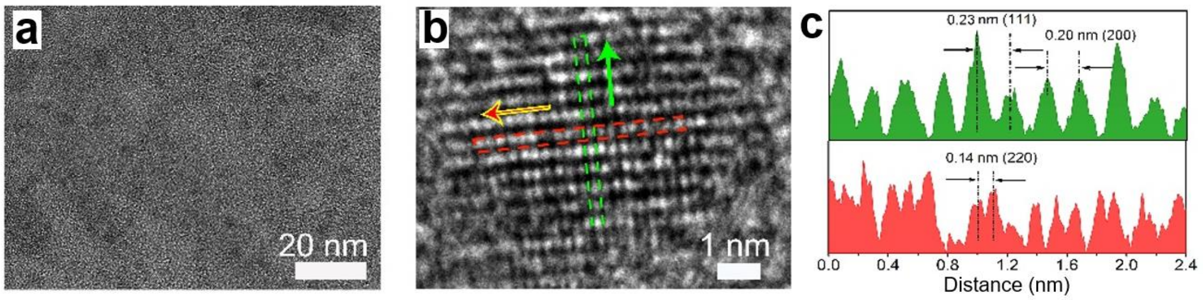

Supplementary Fig. 4 | Morphological characterization. a Low- and **b** high-magnification TEM images of Rh/GDY. **c** Intensity profiles of the olive and red dashed boxes indicated from **b**.

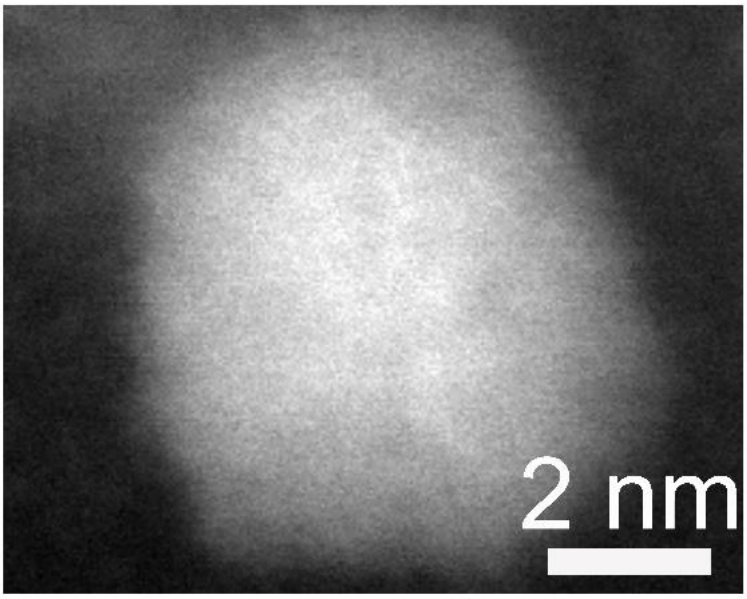

Supplementary Fig. 5 | Morphological characterization. STEM image of Rh/GDY.

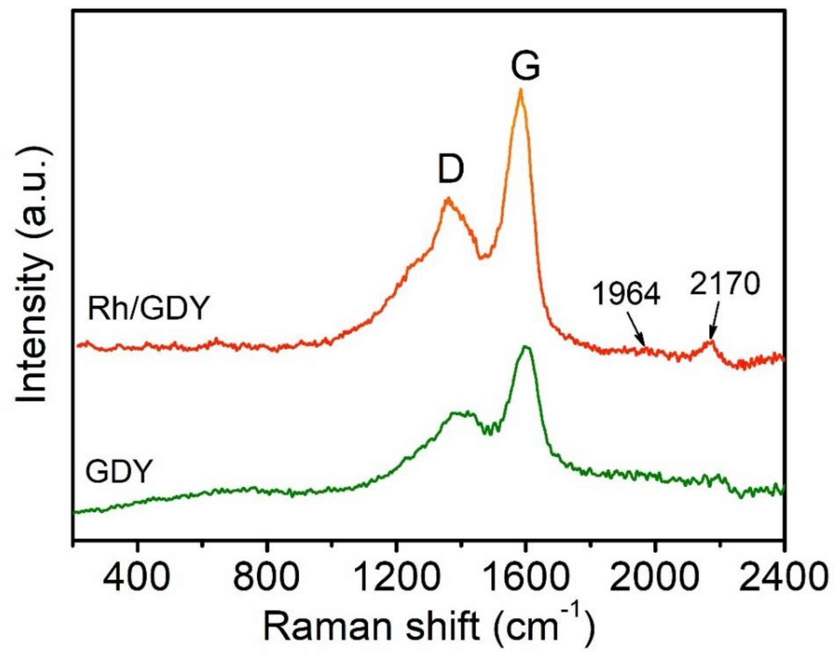

Supplementary Fig. 6 | Structural characterization. Raman spectra of GDY (green line) and Rh/GDY (red line).

Raman spectra of Rh/GDY exhibits four peaks corresponding to the D band (1365 cm^{-1}), G band (1585 cm^{-1}) and vibrations of the conjugated diyne links (1964 cm^{-1} and 2170 cm^{-1}). The relatively higher intensity ratio of D band and G band (I_D/ I_G = 0.63) for Rh/GDY than that of (I_D/ I_G = 0.55) for GDY indicates much more defects existing inside to generate more active sites, which was beneficial for enhancing catalytic performance.

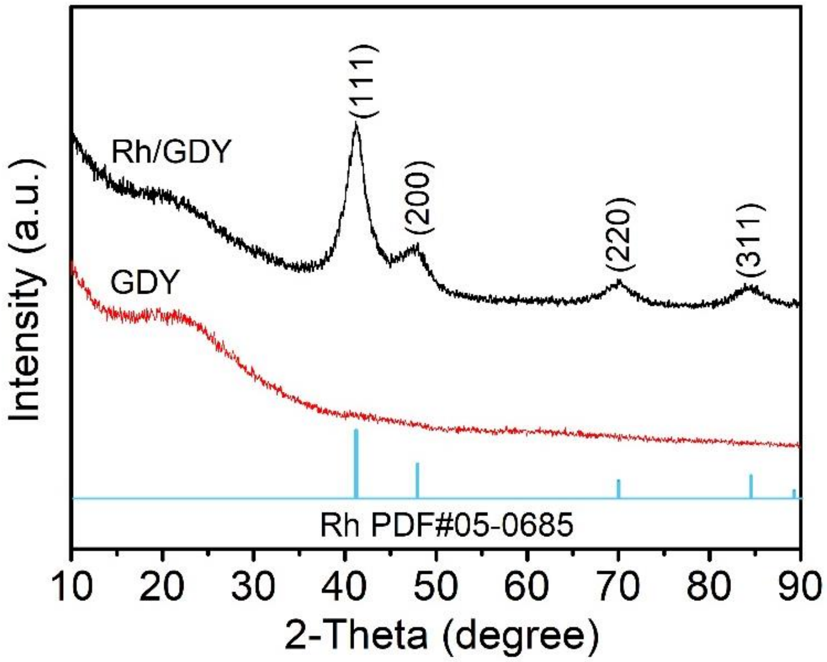

Supplementary Fig. 7 | Structural characterization. XRD spectra of GDY and Rh/GDY.

The XRD pattern shows the diffraction patterns of Rh/GDY at around 41º, 48º, 70º and 84º could be assigned to the metallic Rh (JCPDS no. 05-0685) and two broad diffraction peaks centered at 20-30° and 44° could be attributed to GDY.

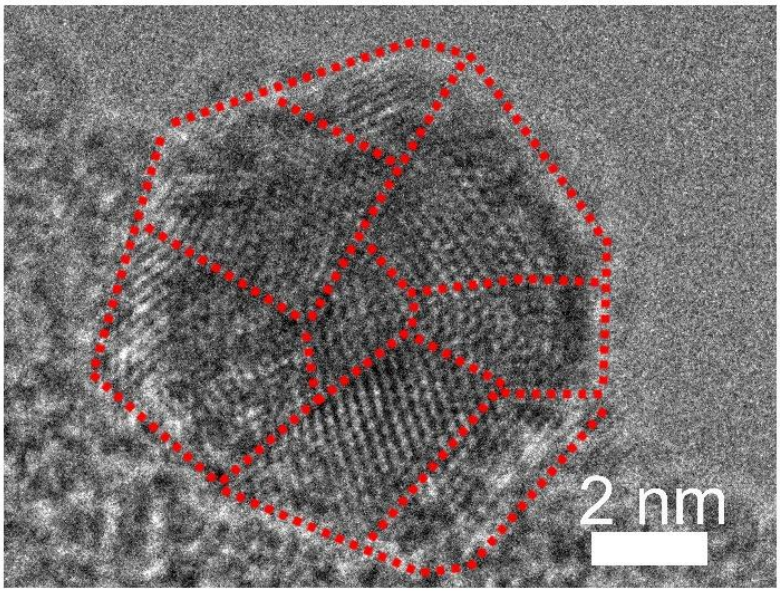

Supplementary Fig. 8 | Morphological characterization. High-resolution TEM image of a single Rh nanocrystal grown on GDY. The labelled red dotted line indicated the axis of the polyhedral nanocrystal.

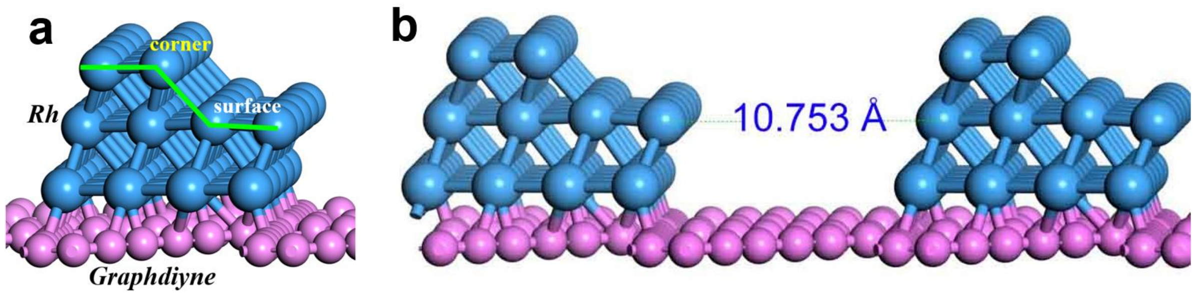

Supplementary Fig. 9 | Structure models. a The stepped surface model of Rh/GDY. **b** The periodic supercell model of stepped Rh/GDY. The distance between the two nearest Rh cluster is 10.753 Å, which is large enough to avoid the interaction.

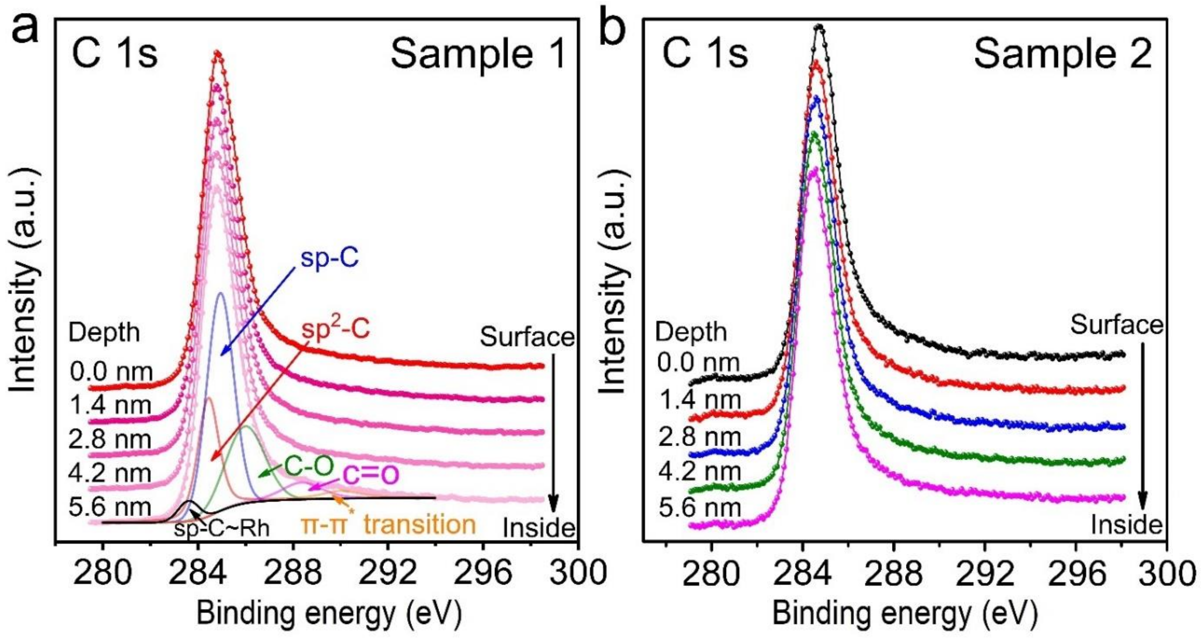

Supplementary Fig. 10 | XPS measurements. XPS depth profiling of C 1s region with different etching depth obtained from **a** sample 1 and **b** sample 2.

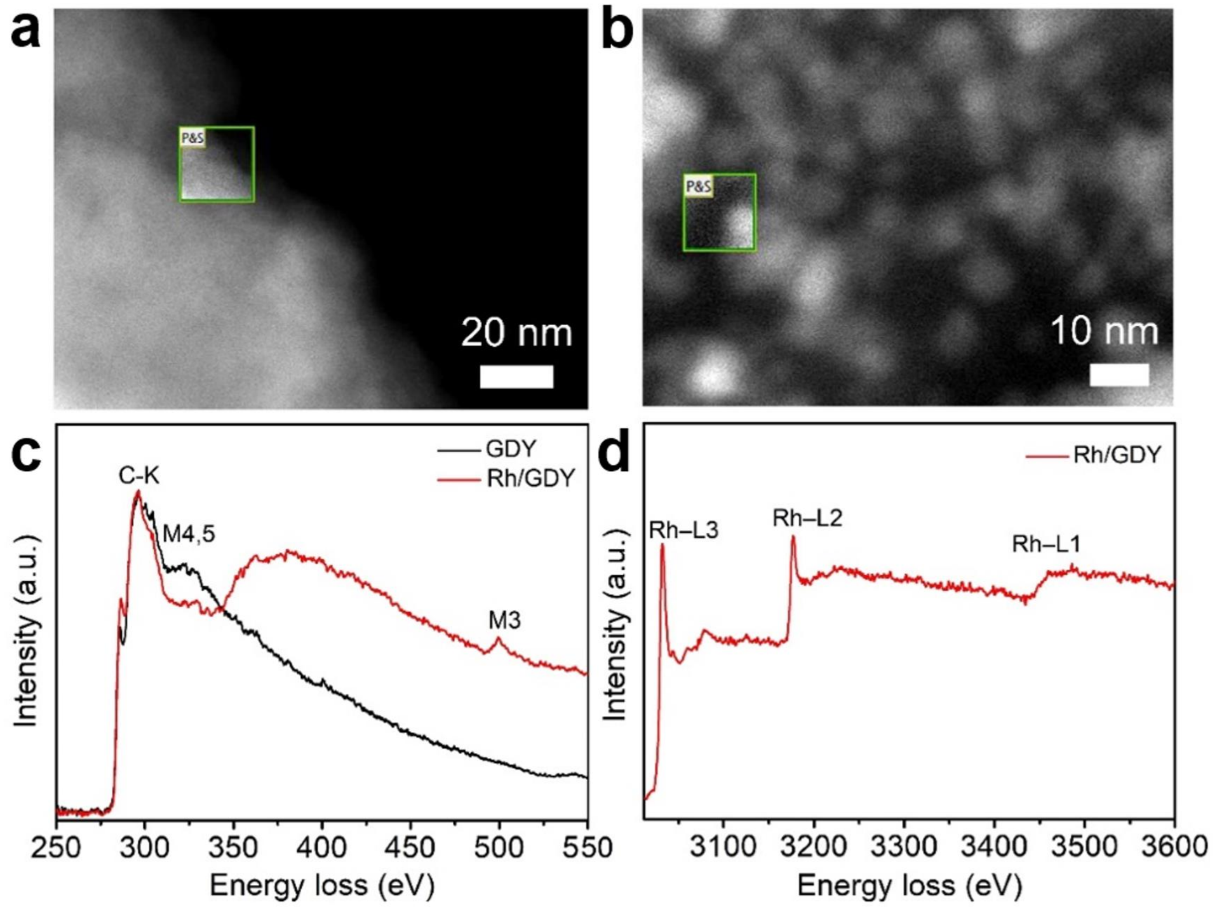

Supplementary Fig. 11 | EELS measurements. EELS scanning area of **a** GDY and **b** Rh/GDY. **c** The results of carbon element in GDY and Rh/GDY form (**a**) and (**b**). **d** The results of rhodium element in Rh/GDY from (**b**).

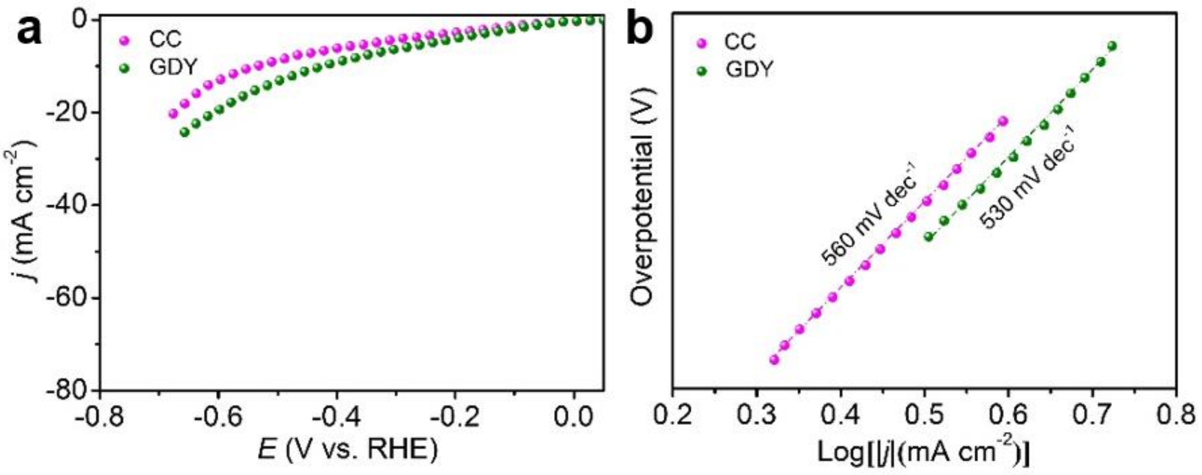

Supplementary Fig. 12 | Electrocatalytic tests. a Polarization curves and **b** corresponding Tafel slopes of the catalysts for HER in saline water (1.0 M KOH + 0.5 M NaCl).

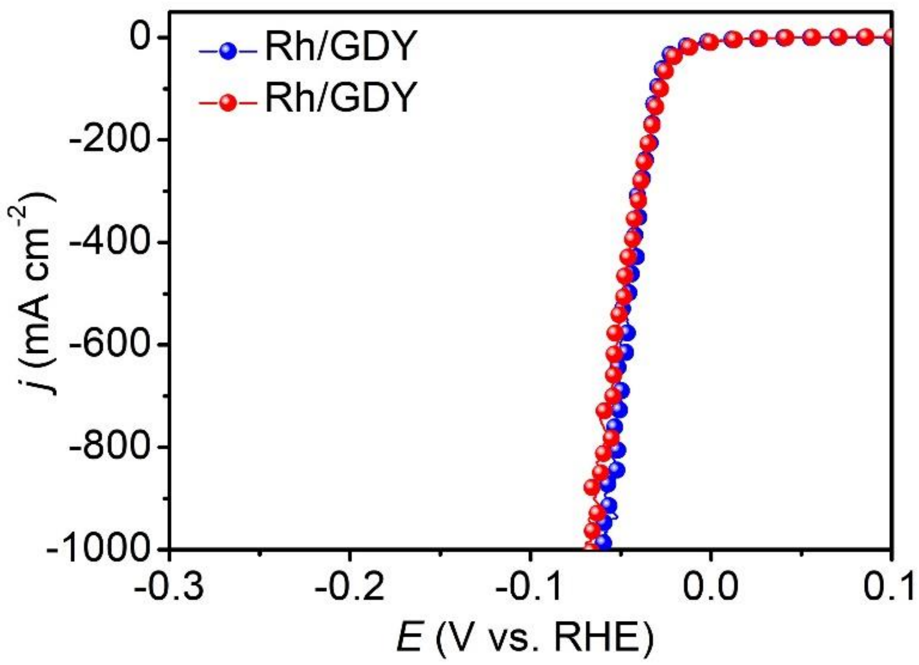

Supplementary Fig. 13 | Electrocatalytic tests. Polarization curves of Rh/GDY for HER in 1.0 M KOH (blue dotted line) and saline water (1.0 M KOH + 0.5 M NaCl; red dotted line).

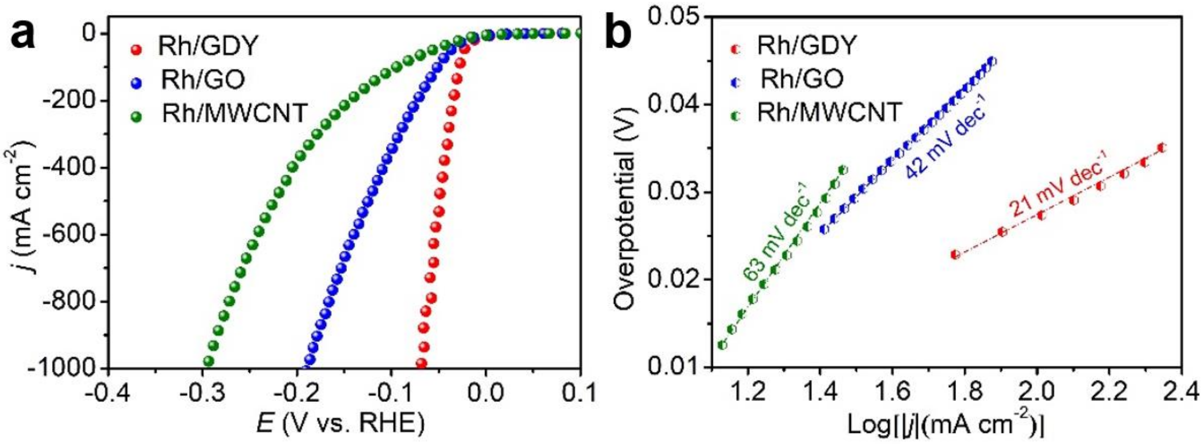

Supplementary Fig. 14 | Electrocatalytic tests. a Polarization curves and **b** corresponding Tafel slopes of Rh/GDY, Rh nanocrystals supported on graphene oxide (Rh/GO) and Rh nanocrystals supported on multi-walled carbon nano-tube (Rh/MWCNT) for HER in saline water (1.0 M KOH + 0.5 M NaCl).

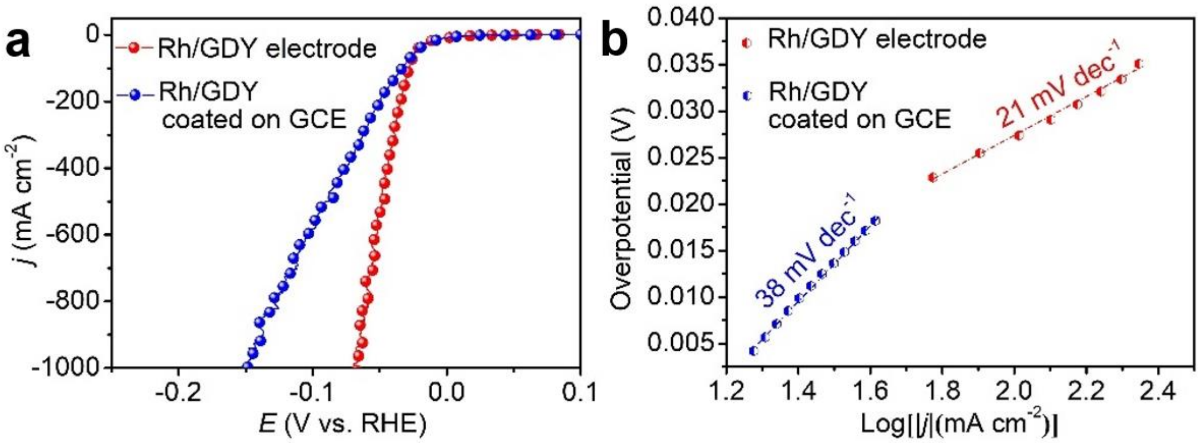

Supplementary Fig. 15 | Electrocatalytic tests. a Polarization curves and **b** corresponding Tafel slopes of Rh/GDY electrode and Rh/GDY coated on glassy carbon electrode (GCE) for HER in saline water (1.0 M KOH + 0.5 M NaCl).

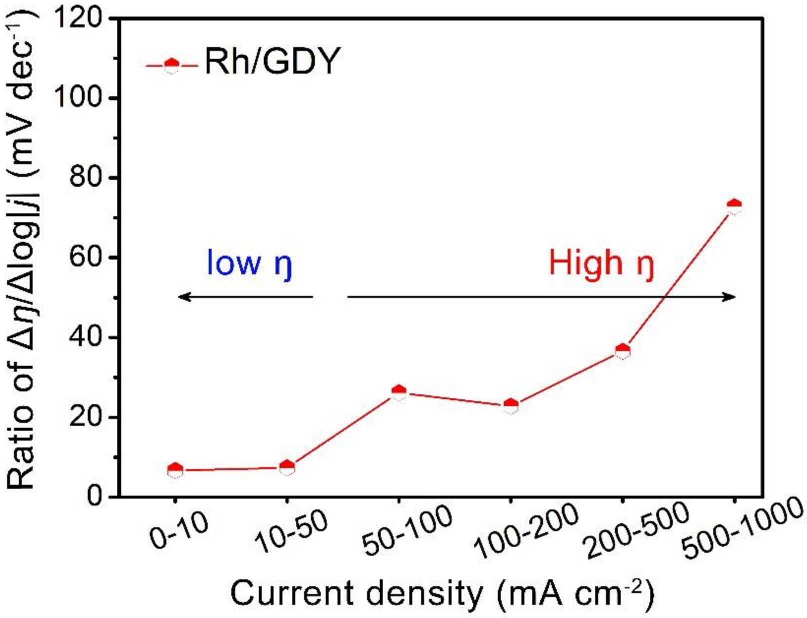

Supplementary Fig. 16 | The ratios of overpotential to current density. Ratios of $\Delta\eta/\Delta\log|j|$ ($R_{\eta/j}$) for Rh/GDY in different current density ranges, which can be used as an indicator to evaluate the performance of a catalyst at high current densities.

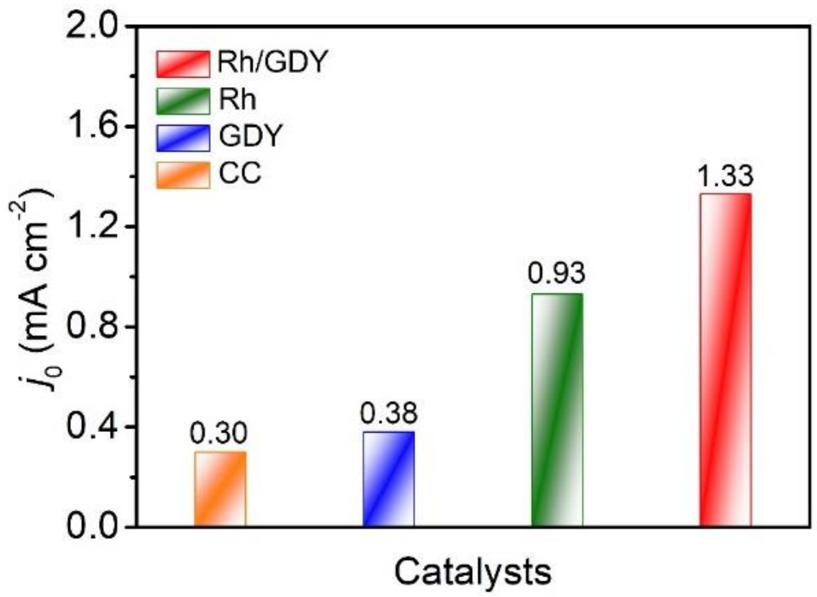

Supplementary Fig. 17 | Electrocatalytic performances. The exchange current density (j_0) of the catalysts for HER in saline water (1.0 M KOH + 0.5 M NaCl).

The j_0 of Rh/GDY was 1.33 mA cm^{-2}, which was significantly higher than that of Rh (0.93 mA cm^{-2}), GDY (0.38 mA cm^{-2}) and CC (0.30 mA cm^{-2}).

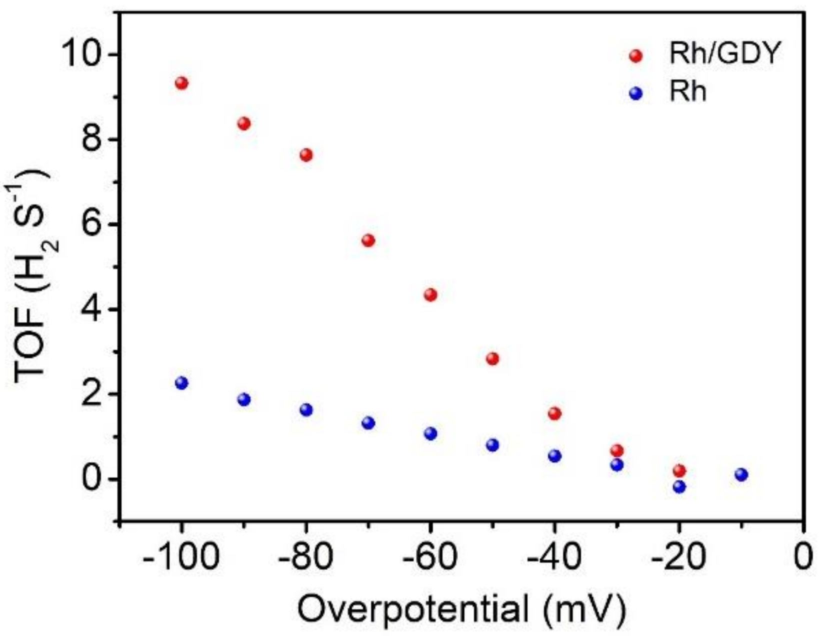

Supplementary Fig. 18 | TOF results. The TOF values of Rh/GDY and Rh as a function of overpotential.

At an overpotential of 100 mV, the TOF values of Rh/GDY and Rh were 9.33 s^{-1} and 2.26 s^{-1}, respectively.

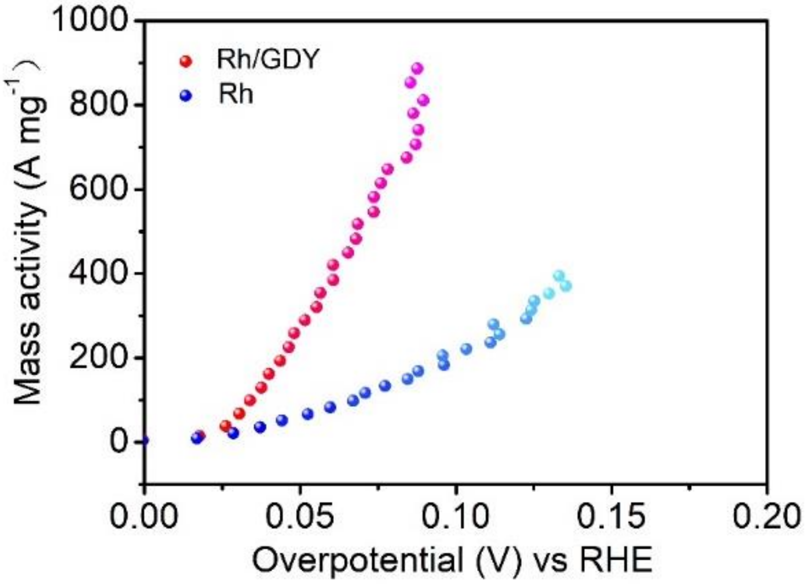

Supplementary Fig. 19 | Electrocatalytic performances. Mass activity of Rh/GDY and Rh.

The mass activity of Rh/GDY and Rh were calculated according to the catalyst active sites. At an overpotential of 50 mV, the mass activities of Rh/GDY and Rh were 274.6 A mg_{Rh}^{-1} and 62.3 A mg_{Rh}^{-1}, respectively.

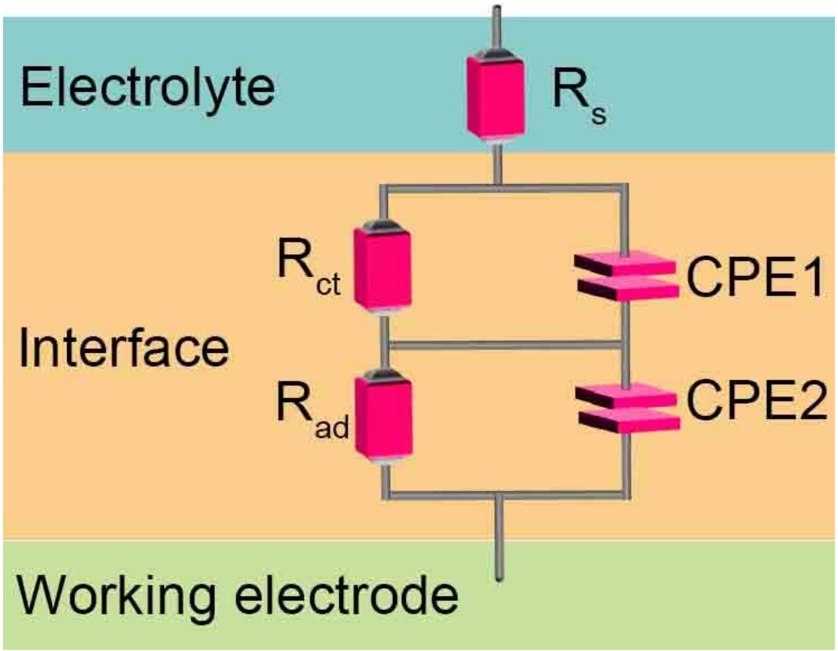

Supplementary Fig. 20 | EIS fitting model. The R(QR)(QR) equivalent circuit model.

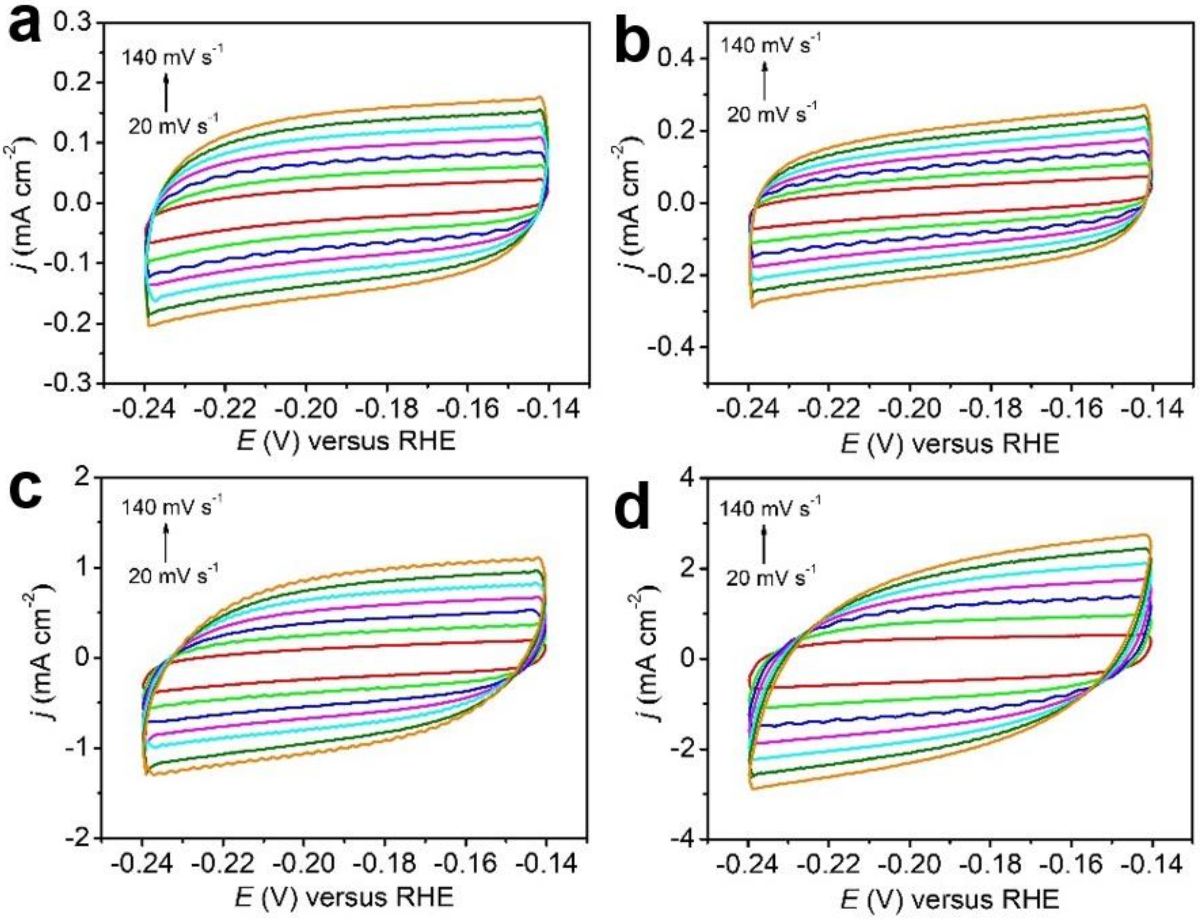

Supplementary Fig. 21 | CV measurements. CV curves of **a** CC, **b** GDY, **c** Rh and **d** Rh/GDY obtained at different scan rates of 20, 40, 60, 80, 100, 120 and 140 mV s^{-1} in saline water (1.0 M KOH + 0.5 M NaCl).

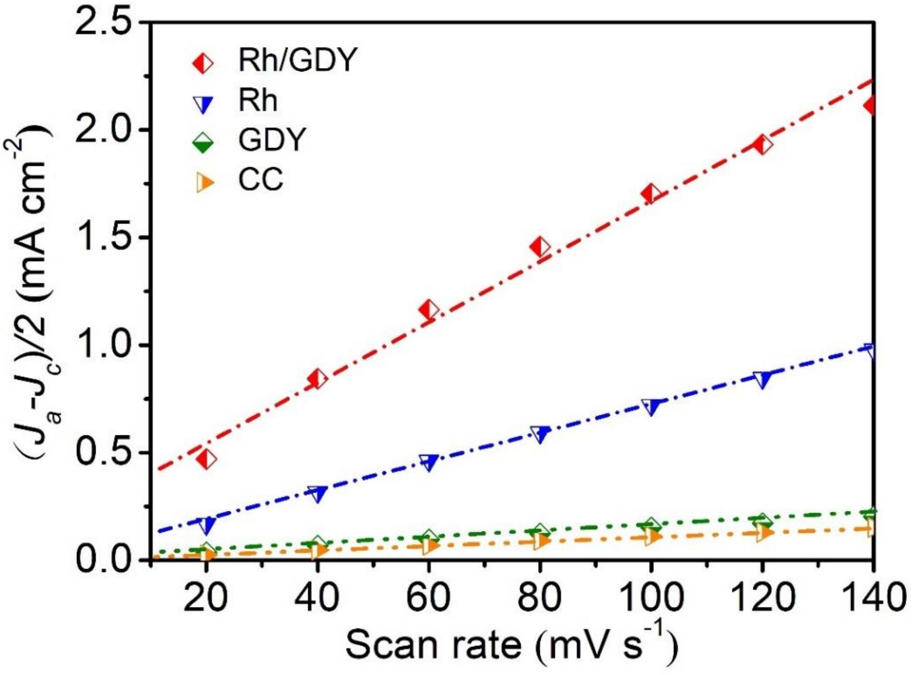

Supplementary Fig. 22 | C_{dl} measurements. The capacitive current density for the catalysts against scan rates in saline water (1.0 M KOH + 0.5 M NaCl).

The C_{dl} of Rh/GDY, Rh, GDY and CC were determined to be 11.8, 6.7, 1.37 and 1.05 mF cm^{-2}, respectively. For calculating the ECSC, we use specific capacitances (C_s) of 0.04 mF cm^{-2}. The ECSA of Rh/GDY was determined to be 295 cm^2, which was greater than that for Rh (167.5 cm^2), GDY (34.3 cm^2) and CC (26.3 cm^2). These results confirmed the largest amounts of the active sites for Rh/GDY, which benefits to the electrocatalytic activity.

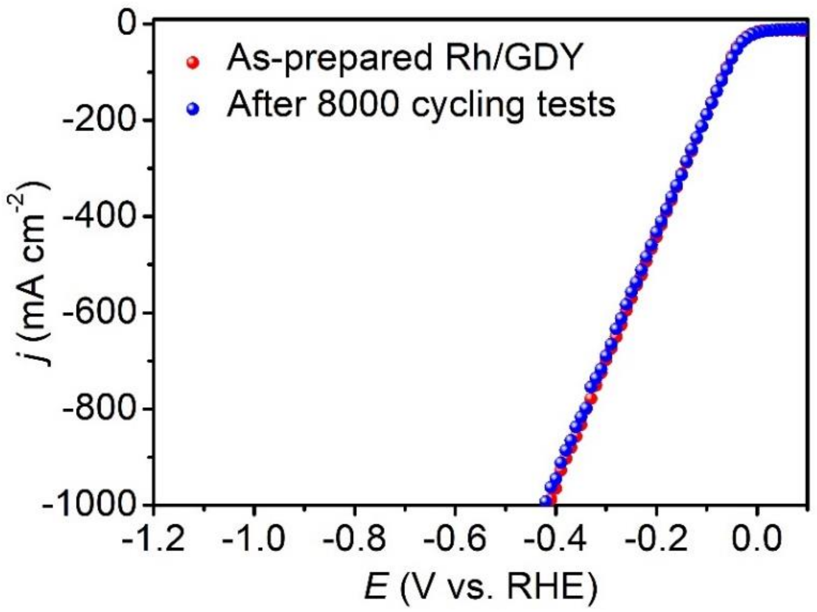

Supplementary Fig. 23 | Stability tests. The LSV polarization curves without *iR*-compensation for the Rh/GDY.

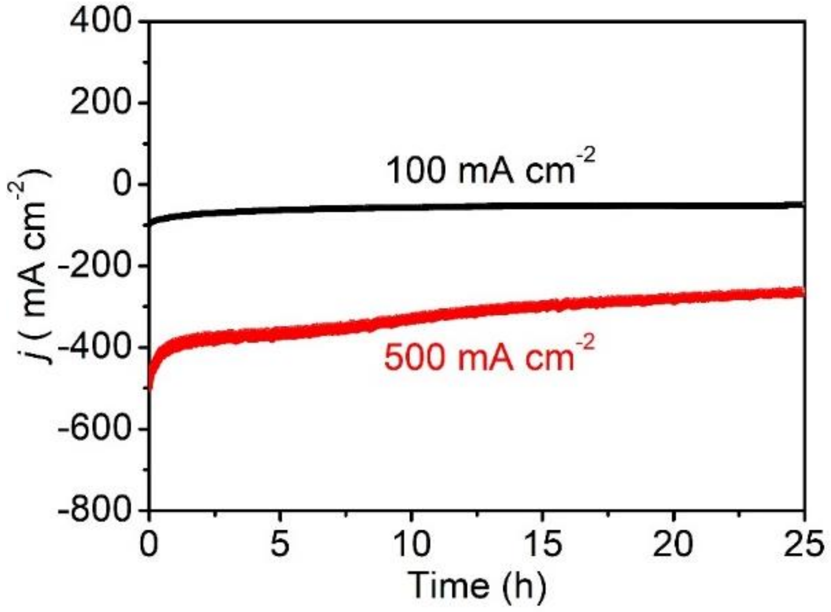

Supplementary Fig. 24 | Stability tests. The current–time (i–t) chronoamperometric response of electrocatalysts at a potential of −0.028 V and −0.048 V (versus RHE) in saline water (1.0 M KOH + 0.5 M NaCl).

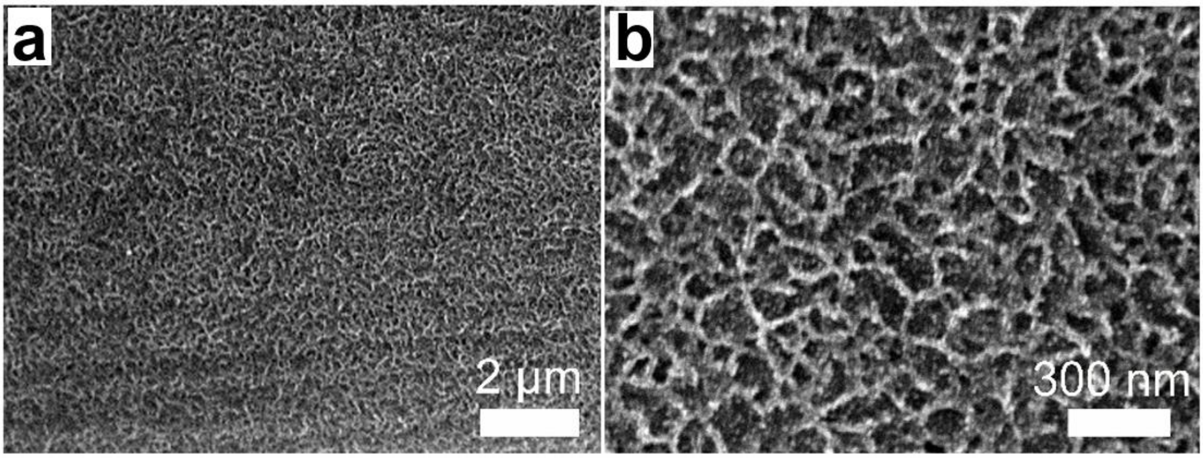

Supplementary Fig. 25 | Morphological characterization. a Low- and **b** high-magnification SEM images of Rh/GDY after continuous cycling test in saline water (1.0 M KOH + 0.5 M NaCl).

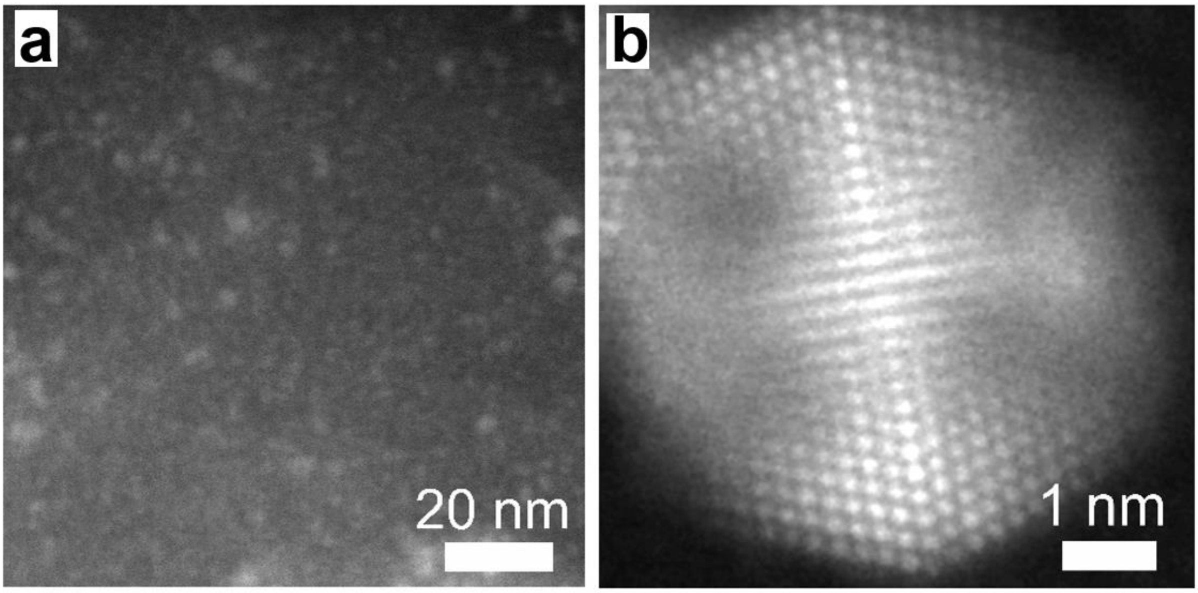

Supplementary Fig. 26 | Morphological characterization. a Low- and **b** high-magnification HAADF-STEM images of Rh/GDY after continuous cycling test in saline water (1.0 M KOH + 0.5 M NaCl).

The HAADF imaging results clearly show that the shape and atomic defects of the Rh/GDY were well maintained during the tests, which confirm the high stability of the catalyst.

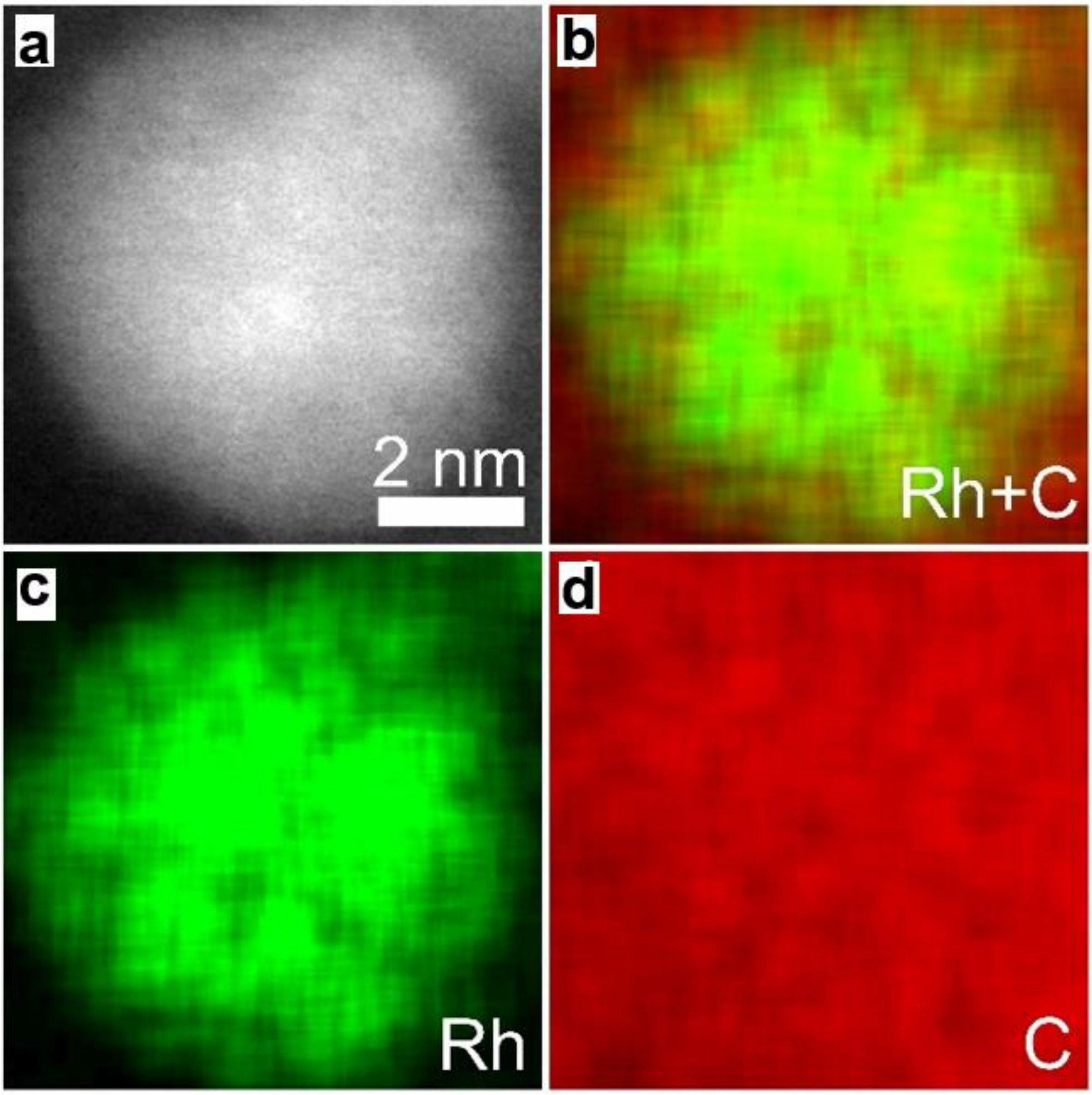

Supplementary Fig. 27 | Elemental mapping. a STEM and **b** overlapping images of Rh/GDY and EDS elemental mapping images of Rh/GDY for **c** Rh and **d** C elements after continuous cycling test in saline water (1.0 M KOH + 0.5 M NaCl).

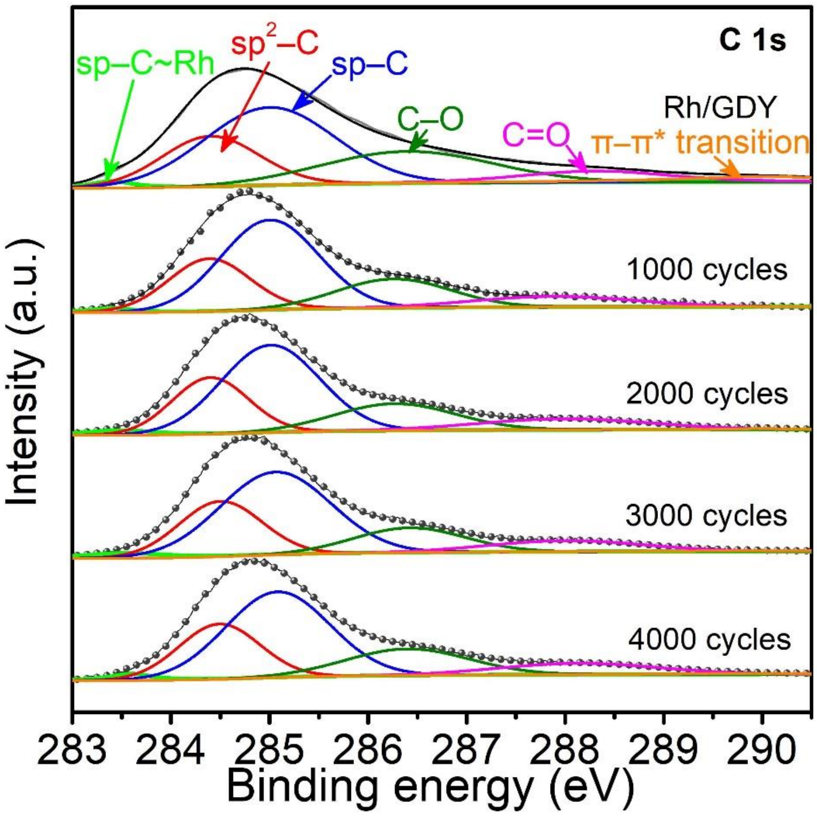

Supplementary Fig. 28 | XPS measurements. C 1s XPS spectra of the Rh/GDY obtained after stability tests.

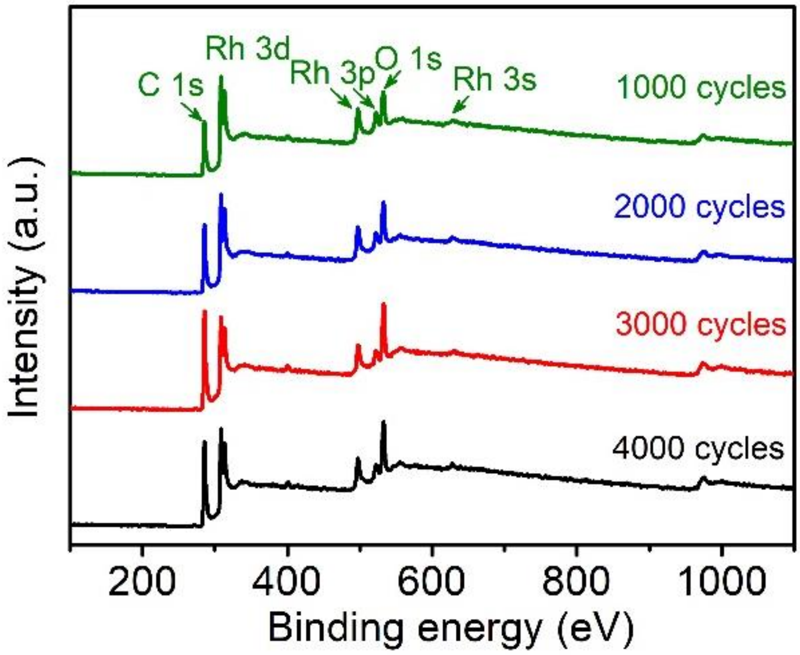

Supplementary Fig. 29 | XPS measurements. The XPS survey spectra of Rh/GDY obtained after stability tests.

There no signals corresponding to the Na^+ and Cl^- species could be observed, which demonstrated the absence of Na^+ and Cl^- on the electrode.

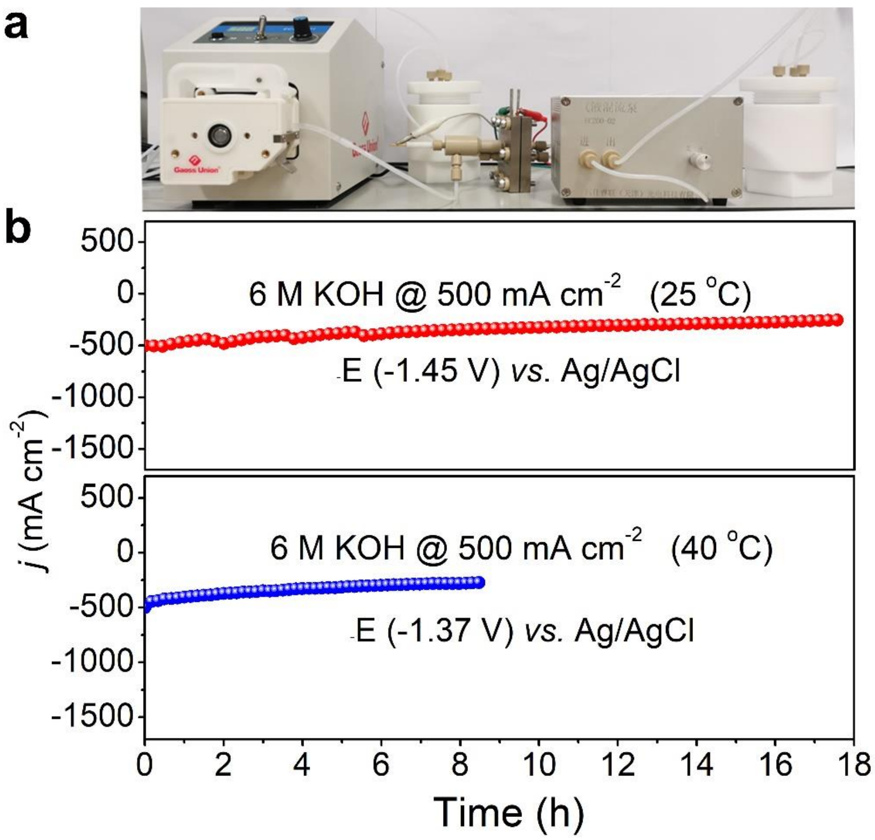

Supplementary Fig. 30 | Stability tests. a Photograph of MEA. b The long-term stability measurements of Rh/GDY by using MEA at 25 °C and 40 °C, respectively.

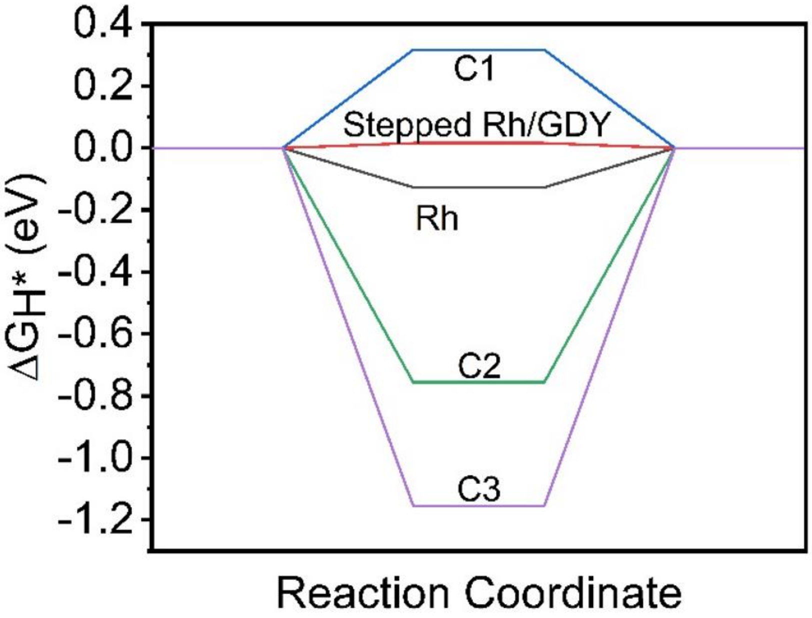

Supplementary Fig. 31 | DFT calculation. Calculated ΔG_{H*} values for Rh and different sites in stepped Rh/GDY.

Supplementary Tables

Supplementary Table 1 | The metal contents of Rh/GDY based on ICP analysis.

**Catalyst**	**Rh (wt%)**
Rh/GDY	0.244
Rh	0.254

Supplementary Table 2 | The overpotentials of all catalyst in saline water (1.0 M KOH + 0.5 M NaCl) to achieve the current density of 10, 100, 500, and 1000 mA cm^{-2}, respectively.

**Catalyst**	***j* (mA cm^{-2})**	***η* (mV)**
CC	10	538
	100	NA
	500	NA
	1000	NA
GDY	10	423
	100	NA
	500	NA
	1000	NA
Pt/C	10	66
	100	NA
	500	NA
	1000	NA
Rh	10	8
	100	55
	500	165
	1000	252
Rh/GDY	10	3
	100	28
	500	48
	1000	65

NA=not available

Supplementary Table 3 | HER activity comparison between the Rh/GDY catalyst and other reported electrocatalysts in different saline water and natural seawater, as well as alkaline electrolytes at room temperature.

Catalysts	Electrolytes	j (mA cm^{-2})	η (mV)@j	Tafel slope (mV dec^{-1})	Ref.
**Rh/GDY**	**1.0 M KOH +0.5 M NaCl**	**10**	**4**	**21**	**This work**
		**100**	**28**		
		**500**	**48**		
		**1000**	**65**		
Alkaline/alkaline simulated Seawater (saline water)					
Ni-SN@C	1.0 M KOH Seawater	10	23	41	Supplementary Ref. 1
Ni-SA/NC	1.0 M KOH Seawater	10	139	123	Supplementary Ref. 2
S, P-(Ni, Mo, Fe)OOH/NiMoP/woo d	1.0 M KOH Seawater	50	187		Supplementary Ref. 3
		100	212		
		200	235		
		500	258		
C-Co_2P	alkaline seawater	1000	192		Supplementary Ref. 4
Ni_2P-Fe_2P/NF	1.0 M KOH +0.5 M NaCl	100	252		Supplementary Ref. 5
		1000	389		
NiMoN@NiFeN	1.0 M KOH +0.5 M NaCl	100	82		Supplementary Ref. 6
		500	160		
		1000	218		
Alkaline Water					
Ru_1/D-NiFe LDH	1.0 M KOH	10	18		Supplementary Ref. 7
		100	61		
Ru-M_xC@CNT	1.0 M KOH	10	15	26	Supplementary Ref. 8
		500	56		
		1000	78		
Pt_{SA}-NiO/Ni	1.0 M KOH	10	26	27	Supplementary Ref. 9
		100	85		
CF/VMFP	1.0 M KOH	10	43	25.2	Supplementary Ref. 10
		100	127		
Cr-Ni NHs	1.0 M KOH	10	75	72	Supplementary Ref. 11
		100	234		
MoC-Mo_2C	1.0 M KOH	500	292		Supplementary Ref. 12
NiCo@C/MXene/CF	1.0 M KOH	10		54.2	Supplementary Ref. 13
		500	235		

β-$NiMoO_4$	1.0 M KOH	10	23	44	Supplementary Ref. 14
		500	210		
MoS_2/Mo_2C	1.0 M KOH	10	87	44	Supplementary Ref. 15
		100	149		
		1000	220		
		100	127		
Ni_2P/NF	1.0 M KOH	10	~58	76	Supplementary Ref. 16
		100	~136		
		1000	306		
FeP/Ni_2P	1.0 M KOH	10	14	24.2	Supplementary Ref. 17
		100	~138		
		1000	~265		
$NiMoO_x$/NiMoS	1.0 M KOH	10	38	38	Supplementary Ref. 18
		100	89		
		500	174		
		1000	236		

Supplementary Table 4 | Comparison of exchange current density (j_0) of Rh/GDY with recently reported catalysts in alkaline solution.

**Catalysts**	**Electrolyte**	j_0 **(mA cm^{-2})**	**Ref.**
**Rh/GDY**	**1.0 M KOH +0.5 M NaCl**	**1.3**	**This work**
W_1Mo_1-NG	1.0 M KOH	0.26	Supplementary Ref. 19
RhPd-H/C	1.0 M KOH	0.65	Supplementary Ref. 20
ES-WC/W_2C	1.0 M KOH	0.58	Supplementary Ref. 21
Sr_2RuO_4	1.0 M KOH	0.898	Supplementary Ref. 22
Ru@CQDs480	1.0 M KOH	0.80	Supplementary Ref. 23
MoC_x nano-octahedrons	1.0 M KOH	~0.029	Supplementary Ref. 24

Supplementary Table 5 | Comparison of the TOF of HER catalysts in alkaline conditions.

**Catalysts**	**Electrolytes**	**TOF (H_2 S^{-1}) at η = 100 mV**	**Ref.**
**Rh/GDY**	**1.0 M KOH +0.5 M NaCl**	**9.3**	**This work**
Ru_1/D-NiFe LDH	1.0 M KOH	7.66	Supplementary Ref. 7
Ru-$Cr_{23}C_6$@CNT	1.0 M KOH	9.2	Supplementary Ref. 8
Pt_{SA}-NiO/Ni	1.0 M KOH	5.71	Supplementary Ref. 9
P-NiMoHZ	1.0 M KOH	0.76	Supplementary Ref. 14
FeP/Ni_2P	1.0 M KOH	0.163	Supplementary Ref. 17
$NiMoO_x$/NiMoS	1.0 M KOH	1.97	Supplementary Ref. 18
W_1Mo_1-NG	1.0 M KOH	0.42	Supplementary Ref. 19
Sr_2RuO_4	1.0 M KOH	0.90	Supplementary Ref. 22
CoP@Ni_2P	1.0 M KOH	0.056	Supplementary Ref. 25
Ru@GnP	1.0 M KOH	0.145	Supplementary Ref. 26
Co-NiS_2	1.0 M KOH	0.55	Supplementary Ref. 27
RuNi/CQDs	1.0 M KOH	5.03	Supplementary Ref. 28
$Mo_1N_1C_2$	1.0 M KOH	0.465	Supplementary Ref. 29
$MoNi_4/MoO_{3-x}$	1.0 M KOH	1.13	Supplementary Ref. 30
Ni_5P_4	1.0 M KOH	0.79	Supplementary Ref. 31

Supplementary Table 6 | Comparison of the mass activity of HER catalysts in alkaline condition.

Catalysts	Electrolytes	Mass activity (A mg_{Rh}^{-1})	Ref.
**Rh/GDY**	**1.0 M KOH +0.5 M NaCl**	**18.2 at 20 mV<br>64.4 at 30 mV<br>274.6 at 50 mV**	**This work**
Pt_{SA}-NiO/Ni	1.0 M KOH	20.6 at 100 mV	Supplementary Ref. 9
RP-CPM	1.0 M KOH	18.2 at 200 mV	Supplementary Ref. 32
PtSA-Ni_3S_2@Ag NWs	1.0 M KOH	7.6 at 150 mV	Supplementary Ref. 33

Supplementary Table 7 | Fitting parameter values derived from the analysis of impedance spectra recorded in saline water (1.0 M KOH + 0.5 M NaCl) at 298 K.

Catalysts	R_s [Ω]	Q_1 [Ss^{-n}]	n_1	R_{ct}	Q_2 [Ss^{-n}]	n_2	R_{ad} [Ω]
Rh/GDY	4.71	4.08×10^{-3}	0.74	2.84	0.33	0.61	10.9
Rh	4.93	2.85×10^{-3}	0.93	9.81	0.16	0.36	28.1
GDY	5.70	3.03×10^{-3}	1	22.2	0.02	0.41	108.2
CC	6.51	1.2×10^{-2}	0.59	89.6	1.9×10^{-4}	0.91	25.8

Supplementary Table 8 | The C_{dl}, ECSA and R_f value of catalysts CC, GDY, Rh and Rh/GDY in saline water (1.0 M KOH + 0.5 M NaCl).

**Catalysts**	**C_{dl} (mF cm^{-2})**	**ECSA (cm^{2})**	***R_f***
CC	1.05	26.3	26.3
GDY	1.37	34.3	34.3
Rh	6.7	167.5	167.5
Rh/GDY	11.8	295	295

Supplementary References:

1. Jin, H. *et. al.* Stable and highly efficient hydrogen evolution from seawater enabled by an unsaturated nickel surface nitride. *Adv. Mater.* **33**, 2007508 (2021).
2. Zang, W. *et al.* Efficient hydrogen evolution of oxidized Ni-N_3 defective sites for alkaline freshwater and seawater electrolysis. *Adv. Mater.* **33**, 2003846 (2021).
3. Chen, H. *et. al.* Wood aerogel-derived sandwich-like layered nanoelectrodes for alkaline overall seawater electrosplitting. *Appl. Catal. B: Environ.* **293**, 120215 (2021).
4. Xu, W. *et al.* Electronic Structure modulation of nanoporous cobalt phosphide by carbon doping for alkaline hydrogen evolution reaction. *Adv. Funct. Mater*. **31**, 2107333 (2021).
5. Wu, L. *et al.* Heterogeneous bimetallic phosphide Ni_2P-Fe_2P as an efficient bifunctional catalyst for water/seawater splitting. *Adv. Funct. Mater.* **31**, 2006484 (2021).
6. Yu, L. *et al.* Non-noble metal-nitride based electrocatalysts for high-performance alkaline seawater electrolysis. *Nat. Commun.* **10**, 5106 (2019).
7. Zhai, P. *et. al.* Engineering single-atomic ruthenium catalytic sites on defective nickel-iron layered double hydroxide for overall water splitting. *Nat. Commun*. **12**, 4587 (2021).
8. Wu, X. *et al.* Solvent-free microwave synthesis of ultra-small Ru-Mo_2C@CNT with strong metal-support interaction for industrial hydrogen evolution. *Nat. Commun.* **12**, 4018 (2021).
9. Zhou, K. *et al.* Platinum single-atom catalyst coupled with transition metal/metal oxide heterostructure for accelerating alkaline hydrogen evolution reaction. *Nat. Commun.* **12**, 3783 (2021).
10. Ji, X. *et. al.* Graphene/MoS_2/FeCoNi$(OH)_x$ and graphene/MoS_2/$FeCoNiP_x$ multilayer-stacked vertical nanosheets on carbon fibers for highly efficient overall water splitting. *Nat. Commun*. **12**, 1380 (2021).
11. Kim, J. *et. al.* Tailoring binding abilities by incorporating oxophilic transition metals on 3D nanostructured Ni arrays for accelerated alkaline hydrogen evolution reaction. *J. Am. Chem. Soc.* **143**, 1399 (2021).
12. Liu, W. *et. al.* A durable and pH-universal self-standing MoC–Mo_2C heterojunction electrode for efficient hydrogen evolution reaction. *Nat. Commun.* **12**, 6776 (2021).
13. Sun, F. *et. al.* Energy-saving hydrogen production by chlorine-free hybrid seawater splitting

coupling hydrazine degradation. *Nat. Commun.* **12**, 4182 (2021).

14. Wang, Z. *et. al.* Manipulation on active electronic states of metastable phase β-$NiMoO_4$ for large current density hydrogen evolution. *Nat. Commun*. **12**, 5960 (2021).
15. Luo, Y. *et al.* Morphology and surface chemistry engineering toward pH-universal catalysts for hydrogen evolution at high current density. *Nat. Commun.* **10***,* 269 (2019).
16. Yu, X. *et. al.*"Superaerophobic" nickel phosphide nanoarray catalyst for efficient hydrogen evolution at ultrahigh current densities. *J. Am. Chem. Soc*. **141**, 7537 (2019).
17. Yu, F. *et. al.* High-performance bifunctional porous non-noble metal phosphide catalyst for overall water splitting. *Nat. Commun.* **9**, 2551 (2018).
18. Zhai, P. *et al.* Engineering active sites on hierarchical transition bimetal oxides/sulfides heterostructure array enabling robust overall water splitting *Nat. Commun*. **11**, 5462 (2020).
19. Yang Y. *et al*. O-coordinated W-Mo dual-atom catalyst for pH-universal electrocatalytic hydrogen evolution. *Sci. Adv.* **6**, eaba6586 (2020).
20. Fan, J. *et. al.* Hydrogen stabilized RhPdH 2D bimetallene nanosheets for efficient alkaline hydrogen evolution. *J. Am. Chem. Soc*. **142**, 3645–3651 (2020).
21. Chen, Z. *et al.* Eutectoid-structured WC/W_2C heterostructures: A new platform for long-term alkaline hydrogen evolution reaction at low overpotentials. *Nano Energy* **68**, 104335 (2020).
22. Zhu, Y. *et al.* Unusual synergistic effect in layered Ruddlesden-Popper oxide enables ultrafast hydrogen evolution. *Nat. Commun.* **10**, 149 (2019).
23. Li, W. *et. al.* Carbon-quantum-dots-loaded ruthenium nanoparticles as an efficient electrocatalyst for hydrogen production in alkaline media. *Adv. Mater*. **30**, 1800676 (2018).
24. Wu, H. B., Xia, B. Y., Yu, L., Yu, X.-Y. & Lou, X. W. Porous molybdenum carbide nano-octahedrons synthesized via confined carburization in metal-organic frameworks for efficient hydrogen production. *Nat. Commun.* **6**, 6512–6519 (2015).
25. Jin, M. *et. al.* Hierarchical CoP@Ni_2P catalysts for pH-universal hydrogen evolution at high current density. *Appl. Catal. B: Environ.* **296**, 120350 (2021).
26. Li, F. *et. al.* Mechanochemically assisted synthesis of a Ru catalyst for hydrogen evolution with performance superior to Pt in both acidic and alkaline media. *Adv. Mater.* **30**, 1803676 (2018).

27. Yin, J. *et. al.* Atomic arrangement in metal-doped NiS_2 boosts the hydrogen evolution reaction in alkaline media. *Angew. Chem. Int. Ed*. **58**, 18676 (2019).
28. Liu, Y. *et. al.* A general route to prepare low-ruthenium-content bimetallic electrocatalysts for pH-universal hydrogen evolution reaction by using carbon quantum dots. *Angew. Chem. Int. Ed*. **59**, 1718 (2020).
29. Chen, W. *et. al.* Rational design of single molybdenum atoms anchored on N-doped carbon for effective hydrogen evolution reaction. *Angew. Chem. Int. Ed*. **56**, 16086 (2017).
30. Chen. Y. *et. al.* Self-templated fabrication of $MoNi_4/MoO_{3-x}$ nanorod arrays with dual active components for highly efficient hydrogen evolution. *Adv. Mater*. **29**, 1703311 (2017).
31. Laursen, A. B. *et. al.* Nanocrystalline Ni_5P_4: a hydrogen evolution electrocatalyst of exceptional efficiency in both alkaline and acidic media. *Energy Environ. Sci*. **8**, 1027 (2015).
32. Li, Y. *et. al.* Partially exposed RuP_2 surface in hybrid structure endows its bifunctionality for hydrazine oxidation and hydrogen evolution catalysis. *Sci. Adv*. **6**, eabb4197 (2020).
33. Zhou, K. *et. al.* Atomically dispersed platinum modulated by sulfide as an efficient electrocatalyst for hydrogen evolution reaction. *Adv. Sci*. **8**, 2100347 (2021).
