# Supplementary material for: Rhodium nanocrystals on porous graphdiyne for electrocatalytic hydrogen evolution from saline water
Source: Nat Commun. 2022 Sep 5;13:5227. doi: 10.1038/s41467-022-32937-2 (PMC9445080; doi:10.1038/s41467-022-32937-2)
Supplement: Supplementary file 3 — Description of Additional Supplementary Files [file 41467_2022_32937_MOESM3_ESM.pdf]

Description of Additional Supplementary Files

File Name: Supplementary Movie 1
Description: The video for hydrogen evolution on Rh/GDY (speed up by 10×).
